# Supplementary material for: ACE2 and TMPRSS2 Immunolocalization and COVID-19-Related Thyroid Disorder
Source: Biology (Basel). 2022 Apr 30;11(5):697. doi: 10.3390/biology11050697 (PMC9138641; doi:10.3390/biology11050697)

Supplementary Figure S1. The oral mucosa was stained as a positive control for ACE2 and TMPRSS2 expression. ACE2 (left panel, brown) and TMPRSS2 (right panel, brown) were expressed in both the cytoplasm and nucleus of the cells in the intermediate layer of the buccal mucosa. Scale bar = 50 μm. ACE2, angiotensin-converting enzyme 2; TMPRSS2, transmembrane protease serine.

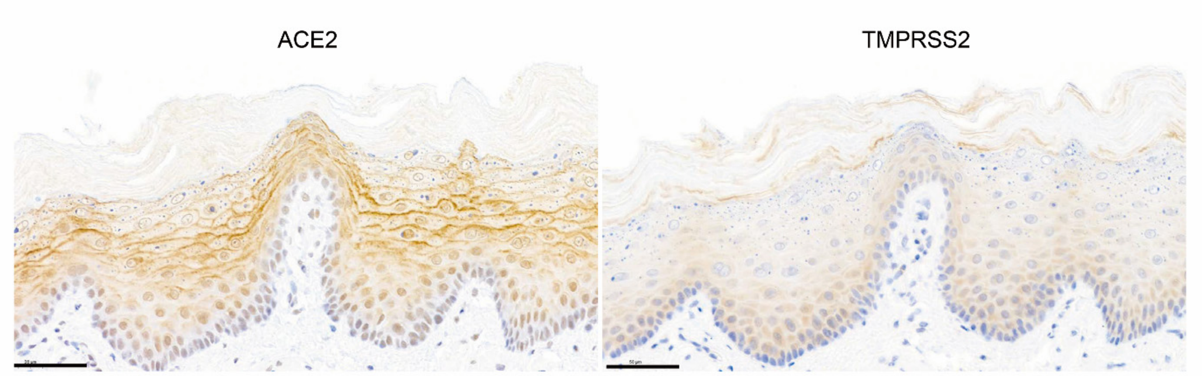

Supplement: Supplementary file 1 [file biology-11-00697-s001.zip › Figure S1.pdf]
