# Supplementary material for: ACE2 and TMPRSS2 Immunolocalization and COVID-19-Related Thyroid Disorder
Source: Biology (Basel). 2022 Apr 30;11(5):697. doi: 10.3390/biology11050697 (PMC9138641; doi:10.3390/biology11050697)
Supplement: Supplementary file 1 [file biology-11-00697-s001.zip › Table S1.pdf]

Supplementary Table S1. Primer sequence used in real-time polymerase chain reaction (PCR).

Gene	Primer sequences
Human	
ACE2	Forward 5′-GGGATCAGAGATCGGAAGAAGAAA-3′
	Reverse 5′-AGGAGGTCTGAACATCATCAGTG-3′
TMPRSS2	Forward 5′-CCTCTAACTGGTGTGATGGCGT-3′
	Reverse 5′-TGCCAGGACTTCCTCTGAGATG-3′
β-actin	Forward 5′-TGCGTGACATGAGAAG-3′
	Reverse 5′-GCTCGTAGCTTCTCCA-3′
GAPDH	Forward 5′-AAATCCCATCACCATCTTCC-3′
	Reverse 5′-GGTTCACACCCATGACGAAC-3
Rat	
ACE2	Forward 5′-ACGAGATGGGACACATCCA-3′
	Reverse 5′-GAAAATCGGATGGCAGAAGA-3′
TMPRSS2	Forward 5′-CACCTGCCATCCACATACAG-3′
	Reverse 5′-CCAGAACTTCCAAAGCAAGC-3′
β-actin	Forward 5′-TGTTGCCCTAGACTTCGAGCA-3′
	Reverse 5′-GGACC CAGGAAGGAAGGCT-3′
GAPDH	Forward 5′-CTGCACCACCAACTGCTTAC-3′
	Reverse 5′-CAGAGGTGCCATCCAGAG-3′
