# Supplementary figures and images for: ACE2 and TMPRSS2 Immunolocalization and COVID-19-Related Thyroid Disorder
Source: Biology (Basel). 2022 Apr 30;11(5):697. doi: 10.3390/biology11050697 (PMC9138641; doi:10.3390/biology11050697)

## <Western blot analysis>

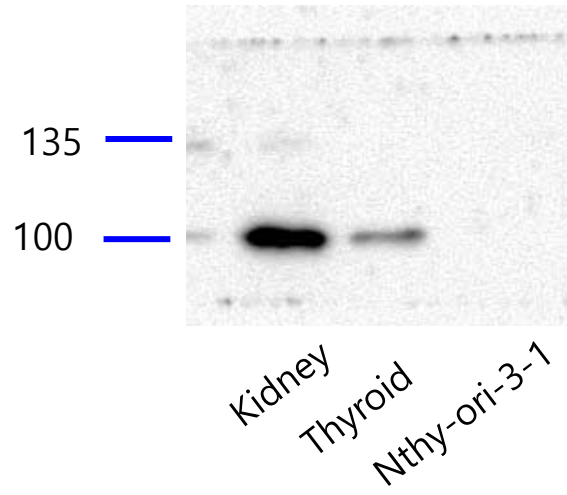

ACE2

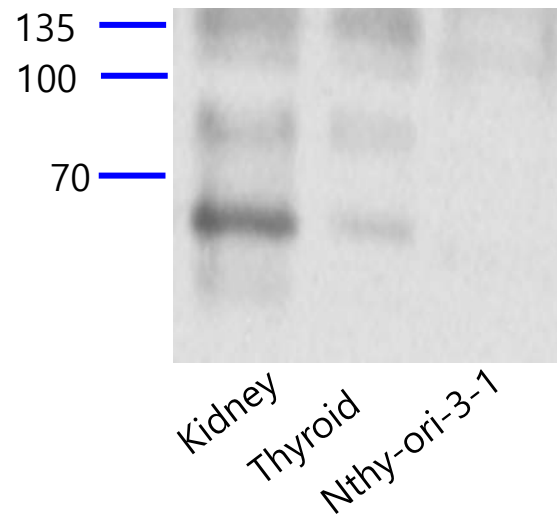

TMPRSS2

Supplement: Supplementary file 1 [file biology-11-00697-s001.zip › File S1.pdf]
